# Total-reflection-based microscope compatible with X-ray and visible light for sample positioning

**DOI:** 10.1107/S1600577526003127

**Published:** 2026-04-14

**Authors:** Kyota Yoshinaga, Jordan Tyler O’Neal, Kai Sakurai, Satoru Egawa, Noboru Furuya, Takenori Shimamura, Yoko Takeo, Yu Nakata, Yuichi Nagayama, Hikaru Kishimoto, Yasunori Senba, Mari Shimura, Taketoshi Kodama, Makina Yabashi, Haruhiko Ohashi, Takashi Kimura

**Affiliations:** a The Institute for Solid State Physics, The University of Tokyo, 5-1-5 Kashiwanoha, Kashiwa, Chiba277-8581, Japan; b RIKEN SPring-8 Center, 1-1-1 Kouto, Sayo-cho, Sayo-gun, Hyogo679-5198, Japan; c Japan Synchrotron Radiation Research Institute 1-1-1 Kouto, Sayo-cho Sayo-gun Hyogo679-5198 Japan; d Research Center for Advanced Science and Technology The University of Tokyo 4-6-1 Komaba Meguro-ku Tokyo153-8904 Japan; e Japan Institute for Health Security, 1-12-1 Toyama, Shinjuku-ku, Tokyo162-8655, Japan; f Graduate School of Agricultural and Life Sciences, The University of Tokyo, 1-1-1 Yayoi, Bunkyo-ku, Tokyo113-8657, Japan; DESY, Germany

**Keywords:** sample positioning, visible light, soft X-ray microscope, Wolter mirror, biological specimen

## Abstract

Sample positioning in X-ray imaging is often technically demanding and involves radiation exposure to the sample. For easier and damage-free positioning, the integration of a visible-light microscope is an effective and widely adopted approach. However, such visible-light microscopes require careful calibration of the objective optic against the X-ray microscope, which introduces additional challenges. To address this issue, we propose a simple total-reflection-based microscope compatible with X-rays and visible light using a Wolter mirror which functions as both X-ray condenser optic and visible-light objective optic. Calibrating our visible-light microscope requires only simple adjustment of an optical lens and three planar mirrors, while no adjustment of the objective optic. We constructed our proposed system for a soft X-ray ptychography system and successfully demonstrated the simple calibration of the visible-light microscope, which was then applied to position a biological sample without delivering a radiation dose.

## Abstract 2

A total-reflection-based microscope compatible with X-rays and visible light for sample positioning without difficult calibration of the system is introduced.

## 1. Introduction

Soft X-ray microscopy can visualize the internal structure of a sample with high spatial resolution, taking advantage of the short wavelength compared with visible light. In particular, light elements have strong absorption and phase shift contrast in the soft X-rays, enabling high spatial resolution observations of materials and biological samples such as graphene (Schultz *et al.*, 2011 ▸) and mammalian cells (Shimamura *et al.*, 2024 ▸). To achieve high spatial resolution, a condenser optic focuses X-ray beams to a micrometre-scale or smaller size onto a region of interest (ROI) on a sample (Müller *et al.*, 2014 ▸; Takeo *et al.*, 2020 ▸).

To position an ROI in the vicinity of focused X-ray beams, visible-light microscope (VLM) systems have been widely employed with soft X-ray microscope (SXM) systems (Guttmann *et al.*, 1993 ▸; Meyer-Ilse *et al.*, 2000 ▸). Positioning with a VLM reduces radiation damage (Kirz *et al.*, 1995 ▸), and, due to the large field-of-view (FOV), positioning is easier and simpler. In these systems, however, the visible-light objective optics were independently mounted within the SXM systems, requiring careful calibration between the VLM and SXM systems (Dehlinger *et al.*, 2020 ▸). If an X-ray condenser optic were repurposed as a visible-light objective optic, one could image a region in the vicinity of the X-ray focal point with visible light avoiding the difficult calibration. Realizing such a system would require an achromatic X-ray optic with high imaging performance.

Here, we propose a simple microscopic system compatible with X-rays and visible light using a rotational total-reflection Wolter mirror (Wolter, 1952 ▸; Hoshino *et al.*, 2004 ▸) for sample positioning in a soft X-ray ptychographic microscope (Pfeiffer, 2017 ▸). In this system, a single Wolter mirror functions as both the X-ray condenser optic in the SXM and the visible-light objective optic in the VLM. Owing to the achromatic specular reflection on the total-reflection Wolter mirror, the object point of the VLM can coincide with the image point of the SXM—that is, the point onto which the X-rays are focused. In addition, a rotational Wolter mirror contributes to high spatial resolution imaging with a large FOV in visible light. This is due to the small off-axis aberration by the satisfaction of Abbe’s sine condition (Chon *et al.*, 2006 ▸) and high numerical aperture by using the entire aperture of the rotational mirror (Mimura *et al.*, 2018 ▸; Egawa, Owada *et al.*, 2019 ▸). We successfully demonstrated the simple calibration of the VLM system, which was then applied to position an ROI in the vicinity of the focused X-ray beams.

## 2. Microscopic system compatible with X-ray and visible light

**Figure 1:**
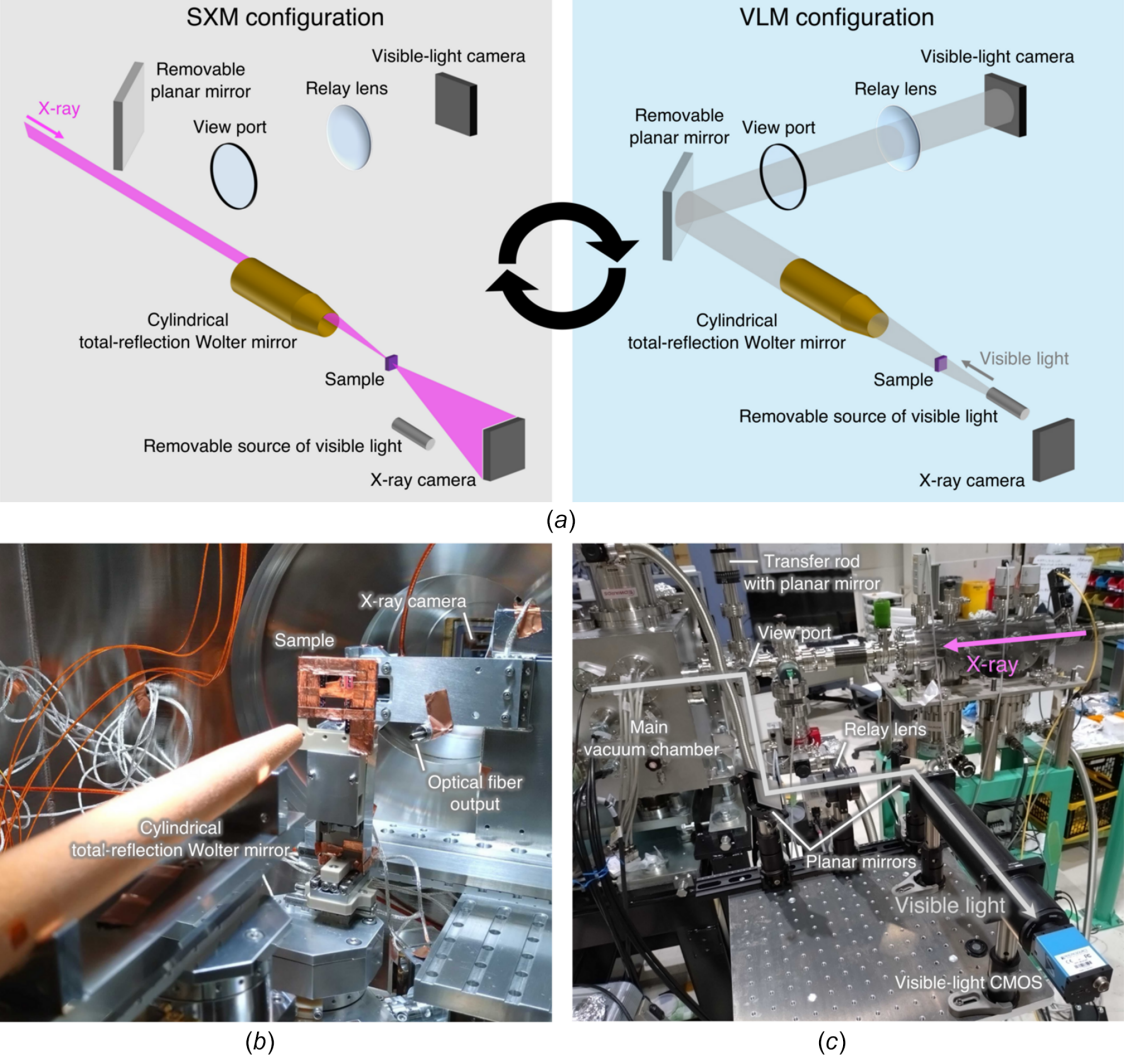
Beamline system in which a single Wolter mirror functions as both X-ray condenser optic in the SXM and visible-light objective optic in the VLM. (*a*) Schematic illustration of both the SXM and VLM configurations. (*b*, *c*) Constructed system (*b*) in the main vacuum chamber and (*c*) out of the vacuum.

Fig. 1 ▸(*a*) shows a concept of our proposed system. In the SXM configuration, incident X-rays illuminate only a lateral sector of the Wolter mirror’s annular aperture and are focused onto a sample before being imaged by an X-ray camera. Conversely, in the VLM configuration, visible light illuminates the sample from the opposite direction of the X-rays. The light can fill the entire Wolter mirror aperture, resulting in a high numerical aperture. A removable planar mirror then reflects the light away from the X-ray beam path and out of the vacuum chamber. To reduce the optical footprint, a relay lens forms the image plane onto a visible-light camera. The two configurations can be readily switched simply by inserting and removing the source of visible light and the planar mirror. In this system, the visible-light objective optic can always form an image of the region surrounding the point where X-rays are focused without realigning the objective optic.

The proposed system was constructed based on our soft X-ray ptychography system (Kimura *et al.*, 2022 ▸) at the soft X-ray beamline BL07LSU, SPring-8, Japan. Figs. 1 ▸(*b*) and 1(*c*) show the main vacuum chamber and the out-of-vacuum VLM components, respectively. Table 1 ▸ shows the optical parameters of the Wolter mirror mounted in the main chamber. The illuminated sectors of the aperture were restricted by a slit in both the SXM and VLM configurations. As the source of visible light, a broadband LED over the wavelength range from 470 to 850 nm (MBB1F1, Thorlabs) was placed out of the vacuum. The visible light was guided through an optical fiber mounted on a stage downstream of the sample inside the main chamber. The optical fiber is inserted into and retracted from the optical path by the stage. A planar mirror was introduced in the X-ray path upstream of the main chamber by using a transfer rod, which reflects the visible light out of the vacuum. The VLM system adds only the optical fiber and the planar mirror into the vacuum. Contamination was hence negligible even in a soft X-ray experiment conducted in high to ultra-high vacuum. In air, the optical path consists of a relay lens with a focal length of 50 cm placed between two planar mirrors mounted on kinematic mounts. The visible-light image was formed approximately 480 mm downstream onto a visible-light CMOS camera (DFK38UX540, ARGO). This design affords a compact footprint despite the long imaging distance of 13.8 m for the Wolter mirror alone due to its grazing angles of several tens of milliradians.

## 3. Experimental result

### 3.1. Calibration and performance of VLM system

Before the VLM system could be implemented, the SXM system was optimized. The VLM system was then calibrated against the SXM. Throughout the calibration process, the Wolter mirror was fixed in place. The VLM calibration procedure, as described below, required only simple adjustment of the relay lens and the three planar mirrors. The transfer rod can roughly adjust the angle of the planar mirror, whereas the kinematic mounts allow for more precise adjustment.

**Figure 2:**
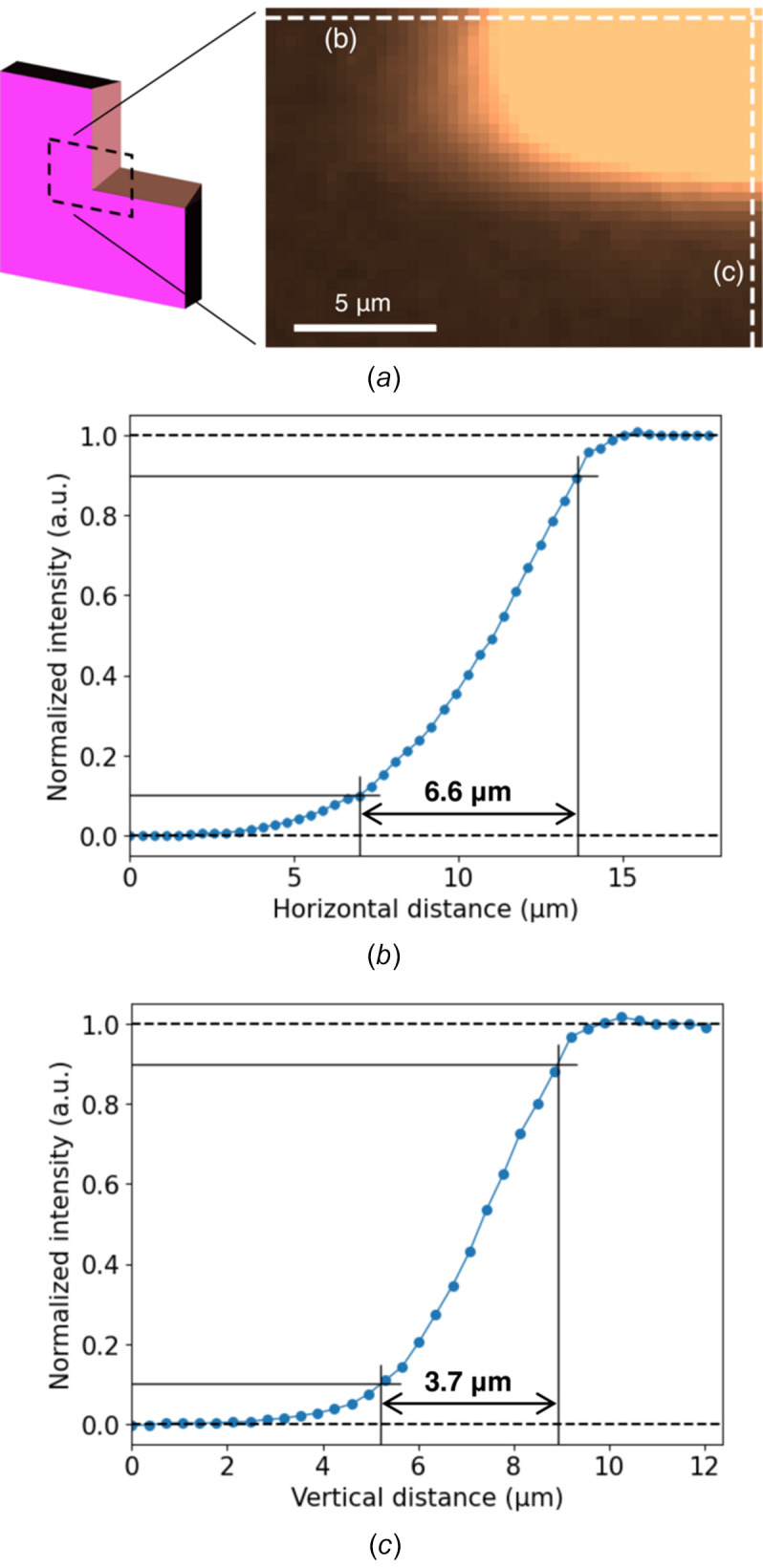
Spatial resolution evaluation using a knife edge. (*a*) Schematic illustration of the knife edge and the VLM image. (*b*, *c*) Intensity lineout along the (*b*) horizontal and (*c*) vertical dashed lines in (*a*).

0. *Precondition: calibration of the SXM system.* The yaw and pitch angles of the Wolter mirror were aligned to find the minimum X-ray focal spot size (Egawa, Yamaguchi *et al.*, 2019 ▸; Kimura *et al.*, 2022 ▸) by scanning a knife-edge corner, shown on the left side of Fig. 2 ▸(*a*). Thereafter, the position of the Wolter mirror was preserved. Since the Wolter mirror has a high tolerance to alignment errors (Egawa, Yamaguchi *et al.*, 2019 ▸; Senba *et al.*, 2020 ▸), the SXM system remains stable throughout the experiments (Sakurai *et al.*, 2025 ▸).

1. *Coarse calibration of the VLM system.* A copper mesh with a large, uniform area was placed approximately near the X-ray focal point. After roughly aligning the relay lens and three planar mirrors by eye, the center of the FOV, at which the maximum spatial resolution is achieved, was brought to the center of the visible-light CMOS sensor with the three planar mirrors.

2. *Fine calibration of the VLM system.* The knife-edge corner was positioned at the X-ray focal point found during the precondition process. The corner was then brought to the center of the visible-light FOV using the two in-air planar mirrors. The position of the relay lens was then adjusted along the optical axis so that the CMOS sensor lay within the depth of focus.

Note that the alignment of the planar mirror on the transfer rod was lost when the mirror was retracted for the SXM configuration due to a low reproducibility of the transfer rod. This issue can be avoided by introducing a motorized transfer rod or a planar mirror with a small aperture in the center through which the X-rays pass.

Upon completion of the coarse calibration, we evaluated the FOV of the VLM system with the copper mesh by counting the number of 26 µm-wide mesh openings visible in the image. The horizontal FOV was estimated to be 1 mm, which was limited by the horizontal sizes of the planar mirrors, whereas the vertical FOV was 1.3 mm, constrained by the size of the visible-light CMOS sensor. After the fine calibration, we evaluated the spatial resolution with the knife edge. Fig. 2 ▸(*a*) shows a diagram of the knife edge with the area observed by the VLM system shown as the dashed rectangle, along with the corresponding VLM image. Figs. 2 ▸(*b*) and 2(*c*) show intensity lineouts taken along the dashed lines in Fig. 2 ▸(*a*). The horizontal and vertical spatial resolutions were estimated to be 6.6 µm and 3.7 µm, respectively, based on the 10–90% distances. Considering the samples ranging from a few to tens of micrometres to which our ptychography system have been applied (Takeo *et al.*, 2023 ▸; Sakurai *et al.*, 2025 ▸), the demonstrated resolution is enough for ROI positioning.

### 3.2. Sample positioning and X-ray imaging

**Figure 3:**
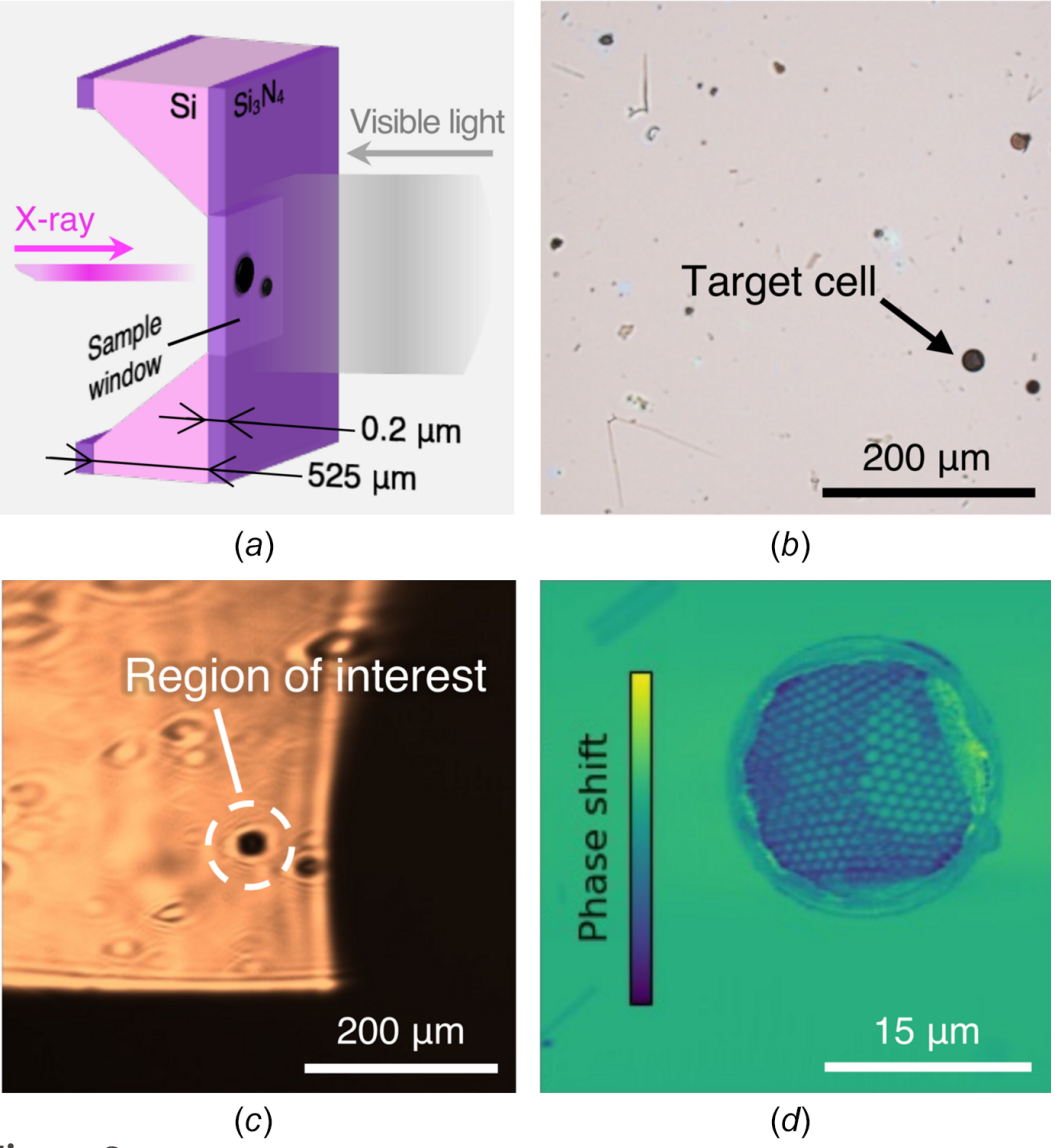
ROI positioning and soft X-ray ptychographic imaging. (*a*) Schematic illustration of a sample chip. (*b*, *c*) Images of the sample window observed with (*b*) a commercial VLM and (*c*) our VLM system. (*d*) Reconstructed phase image of the ROI positioned using our VLM system.

After aligning the SXM and VLM systems, we used the VLM to position an ROI for soft X-ray ptychographic imaging. The target was a single marine diatom cell collected from waters south of Japan. The target cell was placed on a sample chip, the cross section of which is illustrated in Fig. 3 ▸(*a*). The sample chip consisted of a 525 µm-thick silicon layer and 0.2 µm-thick silicon nitride membranes, with the sample placed within a sample window. The knife edge and sample chip were mounted separately on a single sample holder. Fig. 3 ▸(*b*) shows an image of the sample window captured out-of-vacuum before the experiment using a commercial VLM. The target cell is seen as a black circular object located in the lower right area.

Scanning the transverse sample position in our VLM system, the ROI was found and positioned at the center of the FOV. The VLM image is shown as Fig. 3 ▸(*c*). Looking for the ROI is enhanced by the large FOV and few-µm spatial resolution, which allows for easy identification of the target even in the presence of other seawater contaminants. The distortion of the image in the vertical direction is an aberration reported in other microscopes using Wolter mirrors (Hoshino & Aoki, 2006 ▸). At this stage, however, the ROI is not correctly positioned in the longitudinal direction. Due to limited mounting precision, the sample chip and knife edge, against which the VLM system was calibrated, had longitudinal positions offset by up to a few 100 µm. This offset cannot be fully corrected with only the VLM because of the longer depth of field than depth of focus of the SXM. To estimate the offset, we first measured a VLM image centering a corner of the sample window. The system was then switched to the SXM configuration, and we used the corner to find the X-ray focal point. Estimating the offset by the two measurements, we corrected the ROI position without delivering any radiation dose to the target. Previously, we had performed sample positioning by directly irradiating the sample with an X-ray beam to roughly identify the location of an ROI, followed by finer positioning through the acquisition of an X-ray ptychographic image. In our previous experiment on a chemically fixed cell, a single ptychographic measurement resulted in an X-ray dose on the order of several tens of kGy (Sakurai *et al.*, 2025 ▸). By employing our proposed system, the X-ray dose delivered to the ROI prior to X-ray imaging can be reduced by more than several tens of kGy.

Using the corrected ROI position, we performed soft X-ray ptychographic imaging with an X-ray photon energy of 771.7 eV. In order to increase the FOV, the sample was intentionally moved 300 µm downstream, increasing the X-ray beam size to approximately 7.5 µm (horizontal) × 8.9 µm (vertical). The diffraction patterns were measured at 31 × 31 points on the sample with a step size of 1.25 µm, after which the absorption and phase images were reconstructed using the extended ptychography iterative engine algorithm (Maiden & Rodenburg, 2009 ▸). The resulting phase image is shown in Fig. 3 ▸(*d*). The fine structure of the sample was clearly reconstructed. By measuring the distance between the scan center found with the VLM and the actual center of the target cell on the image, the positioning error on the sample plane was determined to be 6.3 µm and 4.3 µm in the horizontal and vertical directions, respectively. The error was comparable with the spatial resolution of the VLM, suggesting that the spatial resolution is the primary cause. We find no evidence of alignment drift during the time between VLM positioning and X-ray ptychographic imaging, and the X-ray ptychography system exhibits small drifts amounting to less than 100 nm h^−1^ (Sakurai *et al.*, 2025 ▸). Our experimental results demonstrated that the proposed system can be effectively employed for sample positioning with an accuracy of a few micrometres in soft X-ray ptychographic microscopy.

## 4. Conclusions and outlook

We developed a microscope system compatible with X-rays and visible light for sample positioning in X-ray microscopy. The calibration requires only the simple adjustment of an in-vacuum planar mirror together with out-of-vacuum optics, repurposing the in-vacuum X-ray condenser optic as the visible-light objective optic without realignment. We then applied this system to sample positioning for soft X-ray ptychographic imaging and demonstrated the effectiveness for robust positioning while limiting the radiation dose. This can be attributed to the high imaging performance owing to the small off-axis aberration and high numerical aperture of the rotational Wolter mirror.

Our concept can be applied not only to X-ray ptychographic imaging as described here but also to various X-ray technologies. Any technique using transmitted X-rays with a rotational Wolter mirror can directly adopt our concept. Changing our visible-light transmission geometry to a reflection geometry would enable sample positioning for technologies which may measure samples of arbitrary thickness, such as X-ray photoelectron spectroscopy (Horiba *et al.*, 2011 ▸). Furthermore, beyond sample positioning, adding fluorescence filters allows for correlative imaging with X-rays and fluorescence (Reinhard *et al.*, 2023 ▸). 

## Figures and Tables

**Table 1:** Optical parameters of the Wolter mirror

Source–focus (image–object) distance	13.8 m
Numerical aperture for SXM / VLM	0.025 rad / 0.15 rad
Illuminated sector of aperture for SXM / VLM	18° / <360°
Obscuration ratio for VLM	0.68
Grazing angle (ellipsoidal surface)	22.1 mrad
Grazing angle (hyperboloidal surface)	42.5 mrad

## Data Availability

The data that support the findings of this study are available from the corresponding authors upon reasonable request.
